# Cost-Effectiveness of Extracorporeal Photopheresis in Patients With Chronic Graft-vs-Host Disease

**DOI:** 10.36469/001c.92028

**Published:** 2024-02-01

**Authors:** Adrian Peacock, Frances C. Dehle, Oscar A. Mesa Zapata, Francesca Gennari, Maro R.I. Williams, Nada Hamad, Stephen Larsen, Simon J. Harrison, Colman Taylor

**Affiliations:** 1 HTANALYSTS, Sydney, Australia; 2 Mallinckrodt Pharmaceuticals, Staines, UK; 3 Department of Haematology St Vincent’s Hospital, Sydney, Australia; 4 St Vincent’s Clinical School, University of New South Wales, Sydney, Australia; 5 School of Medicine, University of Notre Dame, Sydney, Australia; 6 Sydney Medical School, University of Sydney, Sydney, Australia; 7 Institute of Haematology, Royal Prince Alfred Hospital, Sydney, Australia; 8 Clinical Haematology Peter MacCallum Cancer Centre and Royal Melbourne Hospital, Melbourne, Australia; 9 The George Institute for Global Health, Sydney, Australia; 10 The University of New South Wales, Sydney, Australia

**Keywords:** cost-utility analysis, extracorporeal photopheresis, graft-vs-host disease, health technology assessment, cost-effectiveness analysis

## Abstract

**Background:** The mainstay first-line therapy for chronic graft-vs-host disease (cGVHD) is corticosteroids; however, for steroid-refractory patients, there is a distinct lack of cost-effective or efficacious treatment. The aim of this study was to assess the cost-effectiveness of extracorporeal photopheresis (ECP) compared with standard-of-care therapies for the treatment of cGVHD in Australia. The study formed part of an application to the Australian Government to reimburse ECP for these patients.

**Methods:** A cost-utility analysis was conducted comparing ECP to standard of care, which modeled the response to treatment and disease progression of cGVHD patients in Australia. Mycophenolate, tacrolimus, and cyclosporin comprised second-line standard of care based on a survey of Australian clinicians. Health states in the model included treatment response, disease progression, and death. Transition probabilities were obtained from Australian-specific registry data and randomized controlled evidence. Quality-of-life values were applied based on treatment response. The analysis considered costs of second-line treatment and disease management including immunosuppressants, hospitalizations and subsequent therapy. Disease-specific mortality was calculated for treatment response and progression.

**Results:** Over a 10-year time horizon, ECP resulted in an average cost reduction of $23 999 and an incremental improvement of 1.10 quality-adjusted life-years per patient compared with standard of care. The sensitivity analysis demonstrated robustness over a range of plausible scenarios.

**Conclusion:** This analysis demonstrates that ECP improves quality of life, minimizes the harms associated with immunosuppressant therapy, and is a highly cost-effective option for steroid-refractory cGVHD patients in Australia. Based in part on this analysis, ECP was listed on the Medicare Benefits Schedule for public reimbursement.

## INTRODUCTION

Chronic graft-vs-host disease (cGVHD) is an immune-mediated disease resulting from a complex interaction between donor and recipient adaptive immunity.^1–3^ It remains one of the major complications after hematopoietic stem cell transplantation (HSCT) in Australia and is the leading cause of non-relapse mortality in patients surviving more than 2 years.^4,5^ Activated donor T cells attack the tissues of the transplant recipient as antigenic differences cause the immune response to recognize host tissues as antigenically foreign. The resulting inflammatory cytokines cause tissue damage, with the most commonly involved organs including the liver, skin, mucosa, joints, and gastrointestinal tract.

Despite advances in transplant practice, the incidence of cGVHD is increasing worldwide due to the increased use of allogenic HSCT in older recipients and improvements made in treatments after allogenic HSCT, prolonging survival.^4,6,7^ In Australia, allogenic HSCT increased from 488 procedures conducted in 2013 to 614 procedures in 2018.^8^ Of these recipients, approximately 47% develop cGVHD and 34% become refractory to corticosteroids.^9^ Mortality related to cGVHD is estimated to be between 20% and 40% at 3 years, depending on severity.^10–12^

The first-line treatment for cGVHD consists of corticosteroids, which are used to suppress the immune system, including aberrant immunological processes that cause systemic organ damage. However, over 50% of cGVHD patients will fail to respond adequately.^13–15^ Affected patients require long-term use of immunosuppressive drugs associated with the development of severe side effects and low ongoing quality of life that parallel systemic autoimmune diseases.^6,16^ The greater comorbidity burden is associated with higher rates of non-relapse mortality and inferior overall survival.^16^ Options for second-line therapy are numerous, but consensus on the most favorable choice of agent(s) has not been reached, and patients not responding to steroids are at a high risk of death.^17,18^

Extracorporeal photopheresis (ECP) is a leukapheresis-based, immunomodulatory therapy in which a patient’s leukocytes are collected and treated ex vivo with methoxsalen (UVADEX®; Mallinckrodt Pharmaceuticals plc, Bedminster, NJ) injection for extracorporeal circulation and exposure to UV-A light, and then returned to the patient. Integrated, closed ECP systems complete the processes of cell separation, photoactivation of methoxsalen, and reinfusion of the treated cells back into the patient within an automated and fully integrated process.^19^ Extensive published literature for this treatment illustrates that ECP is both safe and effective as a second-line treatment for cGVHD.^20–25^ In a multicenter, randomized controlled trial (RCT) involving 95 patients with corticosteroid-refractory cGVHD, the comparison between ECP combined with standard care and standard care alone demonstrated a statistically significant overall response rate at week 12 for skin manifestations in the ECP group (40%), compared with 10% in the control group (*p* = .002).^21^ In addition to significantly improving clinical response in organ systems affected by cGVHD, ECP-treated patients may be able to reduce their intake of corticosteroids and the serious adverse events associated with the long-term use of these drugs.^21^

The aim of this analysis was to estimate the cost-effectiveness of ECP compared with other second-line treatment options for cGVHD in patients who are dependent, intolerant, or refractory to steroid treatment, from the perspective of the Australian healthcare system.

## METHODS

This economic evaluation was developed as part of Application No. 1651 to the Australian Medicare Services Advisory Committee (MSAC), to establish an item on the Medicare Benefits Schedule (MBS) to subsidize the cost of ECP for the treatment of cGVHD.

### Population

The modeled population was patients with cGVHD following HSCT who were steroid-refractory, steroid-dependent, or steroid-intolerant. The classification of steroid-dependent and/or intolerant and/or refractory cGVHD was based on the National Institutes of Health (NIH) International Consensus and National Comprehensive Cancer Network guidelines for cGVHD.^17,18^ This defined population aligned with clinical management of cGVHD in Australia and RCT evidence.^21,26^

### Intervention

The treatment regimen for ECP was 3 times in the first week, then 2 times per week for weeks 2 to 12, in line with clinical evidence and Australian regulatory authorization.^21,27^ The label permits patients who respond to the initial 12 weeks of therapy to continue with the regimen of 2 treatments every 4 weeks thereafter.^27^ In addition to significantly improving clinical response in organ systems affected by cGVHD, the aim of ECP treatment is to wean patients off immunosuppressant medication.

### Comparator

A treatment survey and structured interviews were conducted to determine the current standard of care (SoC) for cGVHD in Australia (see **Online Supplementary Material**). The survey was designed based on a survey used for a similar study, with input from Australian hematologists.^28^ Few clinicians specialize in treating cGVHD in Australia. For this reason, purposive snowball sampling was used to identify respondents. The survey was hosted on an online platform (SurveyMonkey Inc., San Mateo, CA, USA) and disseminated via email to hematologists and dermatologists with experience in cGVHD working in Australian hospitals. Descriptive analysis of survey results was conducted in Microsoft Excel (Microsoft Corp., Richmond, VA, USA). Seven Australian clinicians were interviewed via face-to-face semi-structured discussions, and responses to the online treatment questionnaire were received from 10 practicing hematologists or dermatologists, all of whom had experience in treating cGVHD. All respondents agreed that prednisone was the main first-line therapy. The survey indicated there were multiple second-line therapies likely to be replaced by ECP, suggesting treatment choice is dependent on physician experience, resource availability, and patient characteristics. The most commonly used second-line therapies for cGVHD were mycophenolate mofetil (44%), tacrolimus (23%), and cyclosporin (33%). Following progression on second-line therapies, the most commonly prescribed third-line therapies were rituximab (41%), mycophenolate mofetil (41%), tacrolimus (13%), and cyclosporin (5%). Respondents noted that these therapies should be added-on to first-line prednisone, with the goal of weaning patients off steroid therapy. Ruxolitinib and ibrutinib were also identified as potential comparators; however, these therapies were not funded by the Australian Government at the time of conducting the analysis. Additionally, rituximab had no subsidized access for cGVHD through the Australian Pharmaceutical Benefits Scheme (PBS); however, it was often funded at the hospital level for later line disease.

### Model Structure

A Markov model was developed in TreeAge Pro (TreeAge Software, Inc., Williamstown, MA, USA) to assess the cost-effectiveness of ECP for patients with treatment refractory cGVHD. The model was developed based on a previously published economic evaluation for ECP for cGVHD^29^ and adapted for the Australian setting using local guidelines for health technology assessment.^30^ Costs and quality-adjusted life-years (QALYs) were accrued at a cohort level for each treatment scenario at 12-weekly cycles over a 10-year time horizon, discounted at 5% per annum. All costs are presented in 2021 Australian dollars (AUD $1 = USD $0.74).

**Figure 1.**
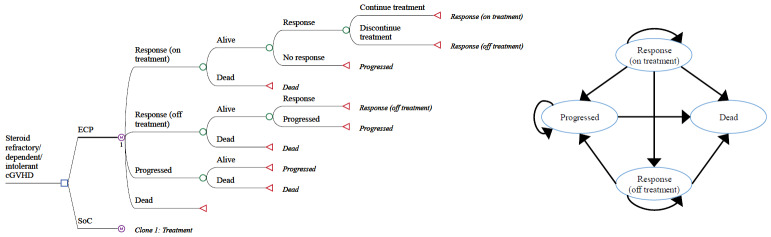
Decision Tree (*left*) and State Transition Diagram (*right*) of the Markov Model Abbreviations: cGVHD, chronic graft-vs-host disease; ECP, extracorporeal photopheresis; SoC, standard of care.

The structure of the economic model is presented in **Figure 1**. Health states were selected and defined based on treatment response as follows: Response (on treatment), response (off treatment), progressed disease, and dead. All patients begin in the response (on treatment) health state. The model applies overall response, discontinuation, progression and mortality rates for ECP and SoC arms at each cycle, which define transitions between health states over time.^21,26^ SoC therapies were modeled collectively based on the available evidence.^21^ Response was differentiated in 2 separate health states by active treatment to recognize the goal for patients to achieve a response and wean them off treatment. Patients that achieve a response at the initial 12-week assessment remain in the response (on treatment) health state, with the probability of transitioning to the response (off treatment), progressed, or dead health states applied at each cycle. Patients in the response (off treatment) health state have the same probability of disease progression and same QALYs as those in the response (on treatment) health state; however, they no longer accumulate treatment costs.

### Clinical Efficacy

Clinical data applied in the economic evaluation included treatment response rate, treatment duration, progression following response, and mortality. Inputs for ECP efficacy and treatment duration were sourced from retrospective registry reviews of adult patients treated with ECP for cGVHD in 2 Australian apheresis centers (Royal Prince Alfred Hospital, Sydney, New South Wales; Peter MacCallum Cancer Centre/Victorian Comprehensive Cancer Centre, Melbourne, Victoria), which were sourced for the registration and reimbursement of ECP in Australia.^26^ Both cohorts examined the safety and efficacy of the integrated, closed CELLEX® Photopheresis System (Therakos, West Chester, Pennsylvania, USA) for steroid-refractory cGVHD in adults with a median follow-up of 4 years.^26^ Patients had moderate to severe cGVHD, a median age of 47 years (range, 20-78 years) and were predominantly male (66%). Acute myeloid leukemia was the most common diagnosis. The baseline characteristics of participants in this study aligned with those in the Australasian Bone Marrow Transplant Recipient Registry.^8^ Data from these treatment centers were selected due to their high external validity and relevance to the Australian setting. In the absence of Kaplan-Meier curves, overall response and mortality rates were calculated assuming a natural logarithmic function over the duration of median follow-up.

Inputs for SoC were sourced from the control arm of the Flowers 2008 RCT, which compared ECP with conventional treatment in patients with treatment refractory cGVHD.^21^ The study was found to be applicable to the studied Australian population, judged on baseline patient characteristics, disease severity, and line of therapy. Treatments used in the control arm of the RCT aligned with those used in Australia, based on responses to the treatment survey.

All variables applied in the economic evaluation are presented in **Table 1**. The probability of disease progression on ECP after 12 weeks was 19.8%.^26^ The probability of disease progression on SoC after 12 weeks was (79.7%) was calculated by applying the relative risk (RR) of complete and partial cutaneous response rates for ECP compared with SoC from the pivotal RCT (RR = 3.95) to the ECP probability.^21^

**Table 1.** Inputs Used in the Economic Analysis

**Variable Description**	**Value**	**PSA Distribution**	**Source**
Transition probabilities			
Treatment response on ECP at week 12	0.802	Beta	MSAC^26^
Disease progression on ECP at week 12	0.198	Beta	
RR of response for SoC compared with ECP at week 12	3.950	Log normal	Flowers, et al,^21^MSAC^26^
Risk of disease progression in responders per cycle after week 12	0.041	Beta	MSAC^26^
Mortality probability for treatment responders per cycle	0.013	Beta	
Mortality probability for non-responders per cycle	0.042	Beta	MSAC^26^^a^
Treatment discontinuation per cycle	0.201	Beta	
Proportion of death due to respiratory failure	0.550	Not varied	MSAC^26^^a^
Utilities			
Response	0.786	Log normal	Crespo, et al^29^
Progressed	0.696	Log normal	Cresp et al^29^
Costs (per cycle)			
ECP	Cycle 1: $52 932 Cycle 2+: $12 704	Not varied	MSAC,^26^Therapeutic Goods Administration^27^
Mycophenolate	$349	Log normal	Department of Health,^31^ Therapeutic Goods Administration^32^
Tacrolimus	$5258	Log normal	Department of Health,^31^ Saad et al^18^
Cyclosporine	$1987	Log normal	Department of Health,^31^ Koc et al^33^
Prednisone	Start dose: $92 Down-⁠titrated dose: $42	Not varied	Department of Health,^31^ MSAC^26^^a^
Subsequent therapy	$5793	Log normal	Department of Health,^31^ Saad et al,^18^ Therapeutic Goods Administration,^32^Koc et al,^33^ Cutler et al^34^
Disease progression	$4324	Log normal	Independent Hospital Pricing Authority^35^
Disease management (treatment response)	$309	Log normal	Department of Health,^36^ expert opinion
Disease management (progressed)	$7147	Log normal	Department of Health,^36^ expert opinion
Hospitalization due to infection	$562	Log normal	Independent Hospital Pricing Authority,^35^ Socié et al^37^
Death from progressive respiratory failure	$21 061	Not varied	Independent Hospital Pricing Authority^35^

All costs presented in 2021 Australian dollars.

^a^Unpublished data.

Abbreviations: ECP, extracorporeal photopheresis; IHPA, Independent Hospital Pricing Authority; MBS, Medicare Benefits Schedule; MSAC, Medicare Services Advisory Committee; PBS, Pharmaceutical Benefits Scheme; PSA, probabilistic sensitivity analysis; RR, relative risk; SoC, standard of care.

Treatment duration following response was calculated from the median maintenance therapy duration in the Australian-specific data^26^ (data unpublished). The median duration of ECP treatment was calculated to be 37 weeks. Treatment duration in the SoC arm of the RCT beyond 12 weeks was not reported.^21^ Given that one aim of treatment is to wean patients off treatment after they achieve a stable response, it was assumed that the probability of discontinuing therapy for responders beyond 12 weeks in the SoC arm was the same as the ECP arm. For those who achieved treatment response at 12 weeks, the probability of disease progression in each subsequent cycle was calculated to be 1%^26^ (data unpublished). The risk of progression in this group was assumed to be the same in the ECP and SoC arms, given the assessment of response is the same regardless of treatment administered. Additionally, it was assumed that the risk of progression was the same in the response (on treatment) and response (off treatment) health states.

### Mortality

Mortality was sourced from the Australian registry review data, which reported mortality of 20% for responders and 53% for non-responders, which aligned with published evidence.^10^ Probabilities were calculated separately for response and progressed health states, assuming a linear function over the study duration.^26^ The study reported that 55% of patients died of progressive respiratory failure. The data demonstrated a higher rate of mortality in patients that were initially assessed as non-responders to ECP treatment compared with patients who responded to treatment, which is consistent with mortality observed in published literature.^37–40^

### Quality of Life

Despite the pivotal RCT demonstrating a significant improvement in quality of life for patients receiving ECP vs SoC as measured by the Targeted Symptoms Assessment,^21^ there is no established method for converting outcomes of the Targeted Symptoms Assessment into utility weights required for economic evaluation. Therefore, quality of life utility values applied in this evaluation aligned with previous economic evaluations,^28,29^ which used studies that measured quality of life of patients with cGVHD using a 30-item questionnaire (EORTC QLQ-C30).^41–43^

### Cost Inputs

Treatment costs, including ECP, SoC, steroid therapy, and subsequent therapy were estimated using prices published on the PBS and published treatment regimens.^27,31,32^ The cost of ECP included the cost of the ECP procedure plus the cost of extracorporeal methoxsalen (UVADEX®; Mallinckrodt Pharmaceuticals plc, Bedminster, NJ, USA). The cost of the ECP service aligned with the fee for ECP for cutaneous T-cell lymphoma on the MBS (MBS item 14249).^26^ The modeled ECP treatment protocol aligned with the clinical evidence and recommended Australian regimen.^21,26,27^ All patients initially continue with steroidal therapy and patients who respond to second-line treatment wean off and receive a lower dose, in line with the goal of second-line treatment. The cost of SoC and third-line subsequent therapy sourced from the PBS was calculated based on the weighting of comparators from the treatment survey.^31^ Disease management costs were calculated for each health state, incorporating unit costs for routine hematological visits and disease monitoring tests as published on the MBS.^36^ Patients in the progressed health state received monthly IV immunoglobulin to manage the impact of immunosuppression as well as increased frequency of monitoring costs and services associated with occupational therapy and mental health assessment. Hospitalization costs considered in the analysis were sourced from published National Hospital Cost Data Collection data.^35^ The cost of hospitalization associated with infection was attributed when patients transition to the progressed health state based on risk of infection.^36,37^ The cost of hospitalization was applied to a portion of those transitioning from the progressed to dead state who died due to progressive respiratory failure.^36^

### Sensitivity Analysis

A deterministic sensitivity analysis was conducted, in line with guidelines for health technology assessment in Australia. Multiple different scenarios were examined during the reimbursement evaluation process, including selection and weighting of SoC and subsequent therapies, time horizon, risk of disease progression, response rate, treatment discontinuation, and mortality.

Although probabilistic sensitivity analyses (PSA) are not a requirement for the economic evaluation of medical technologies in Australia, an exploratory PSA was conducted after the official reimbursement process to align with previously conducted health technology assessment of ECP for cGVHD.^29^ Statistical analyses were made based on published guidance and precedence.^29,44^ Australia does not have a defined willingness to pay (WTP) threshold. A theoretical WTP of $50 000 was selected from analyses of historical reimbursement decisions.^45,46^

## RESULTS

### Primary Analysis

In the base case scenario, ECP was found to be less costly and more effective in terms of QALY compared with SoC for the treatment of steroid-refractory or steroid-dependent or steroid-intolerant cGVHD. Including ECP as a treatment strategy resulted in an incremental cost reduction of $23 999 and an incremental effectiveness improvement of 1.10 QALY (**Table 2**).

**Table 2.** Results of the Cost-Utility Analysis (Per Patient)

**Treatment Arm**	**Cost**	**Incremental Cost of ECP**	**QALY**	**Incremental QALY of ECP**	**ECP ICER**
ECP	$180 020	-$23 999	4.31	1.10	Less costly, more effective
SoC^a^	$204 019		3.21		

All costs presented in 2021 Australian dollars.

^a^Mycophenolate, 44%; tacrolimus, 23%; cyclosporin, 33%.

Abbreviations: ECP, extracorporeal photopheresis; ICER, incremental cost-effectiveness ratio; QALY, quality-adjusted life-year; SoC, standard of care.

Patients in the ECP arm remained on treatment for a longer duration, experienced 2.95 times slower disease progression, and had 20% lower mortality relative to patients in the SoC arm. The Markov traces for ECP and SoC are shown in **Figure S1** and **Figure S2**, respectively. The transition of patients between health states in the model was found to be consistent with the clinical evidence.^26^ ECP delayed initiation of subsequent treatment, reduced utilization of healthcare resources associated with adverse events and disease-related complications including immunosuppression, leading to cost savings.

### Sensitivity Analysis

**Figure 2.**
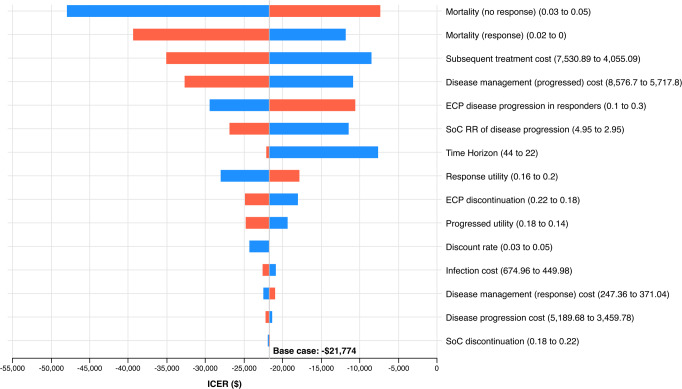
One-Way Sensitivity Analysis: Extracorporeal Photopheresis vs Standard of Care *Blue bars* indicate lower value applied; *red bars* indicate higher value applied. All costs presented in 2021 Australian dollars. Abbreviations: ECP, extracorporeal photopheresis; ICER, incremental cost-effectiveness ratio; RR, relative risk; SoC, standard of care; WTP: willingness to pay.

The results of the deterministic sensitivity analysis showed that the model was sensitive to mortality rate, cost of subsequent treatment and disease management costs (**Figure 2**). However, ECP remained the least costly and most effective treatment strategy in all scenarios tested. Assuming a smaller difference in mortality between responders and non-responders favored ECP, as more patients in the SoC arm remain in the no response health state and continue to accrue costs. Reducing ECP disease progression in responders at 12 weeks reduced the number of patients entering the decreasing progression in the ECP arm, thus favoring ECP. Decreasing the time horizon from 10 years (44 12-weekly cycles) to 5 years (22 12-weekly cycles) increased the incremental cost-effectiveness ratio due to the long-term benefit of ECP not being captured. However, ECP remained less costly and more effective in this scenario.

**Figure 3.**
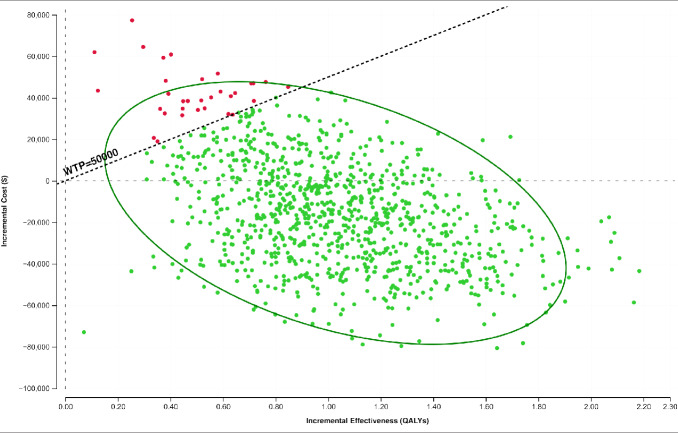
Incremental Cost-Effectiveness of Extracorporeal Photopheresis Probability Sensitivity Analysis Scatterplot *Green plots* indicate cost-effective scenarios using a WTP of $50 000/QALY, *red plots* indicate not cost-effective using a WTP of $50 000/QALY. All costs presented in 2021 Australian dollars. Abbreviation: QALY, quality-adjusted life-year; WTP, willingness to pay.

The exploratory PSA demonstrated that, using a hypothetical WTP threshold of $50 000, ECP was cost-effective in 98% of 1000 iterations (**Figure 3**). The 2.5th and 97.5th percentiles for incremental cost-effectiveness ratio (ICER) were -$45 446 and $74 626, respectively.

## DISCUSSION

This study examined the cost-effectiveness of ECP for treatment of cGVHD in Australia and found ECP to be less costly and more effective than the weighted SoC comparator. Results were driven by the higher response rate of ECP, leading to improved quality of life, reduced immunosuppressant therapy, and decreased disease management and subsequent therapy costs. Our findings were robust as indicated by the results of the PSA.

The results of our analysis align with previous cost-effective analyses demonstrating ECP to be a highly cost-effective treatment option for steroid-refractory cGVHD. A microsimulation conducted from the perspective of the Spanish public payer found ECP to be cost-effective compared with rituximab and imatinib. ECP resulted in a cost per QALY of €8330 compared with rituximab and was less costly and more effective compared with imatinib over a 5-year time horizon.^29^ Similarly, a cost-utility microsimulation from an Italian healthcare perspective found ECP to be less costly and more effective than pentostatin, mycophenolate and imatinib for treatment refractory cGVHD, over a 7-year time horizon.^28^ Our findings are applicable to Australia and align with the results of other cost-effectiveness studies, which found ECP to be a cost-effective treatment regimen compared to other treatment options in their respective jurisdictions.

Previous evaluations have highlighted the lack of high-quality robust clinical evidence for ECP in treating cGVHD.^28^ Our analysis uses Australian-specific ECP efficacy data to demonstrate a significant decrease in costs associated with reduced long-term immunosuppressant therapy, making the findings highly relevant to the Australian setting. The efficacy and safety profile of ECP as a second-line treatment for cGVHD is well established.^47^ Compared with SoC therapies, ECP reduces the use of steroids and other immunosuppressants with poor safety profiles.^20–22,24,26,48^ By partitioning responder health states by treatment use we successfully modeled the goal of reducing immunosuppressant use while remaining free of active disease. There is a significant clinical need for accessible treatments that reduce chronic immunosuppressant use. Patients who are able to lower their daily intake of systemic immunosuppressant medication have reduced risk of adverse effects, including diabetes mellitus, infection, and liver and kidney damage.^49,50^ Furthermore, immunosuppressants, including tacrolimus, cyclosporine and mycophenolate, can inhibit the graft-vs-leukemia effect, thereby increasing relapse rate, requirement for additional immunosuppressants and delaying immune reconstitution.^51–55^

Markov models are preferred for standard health economic assessment as they offer a pragmatic balance between speed and accuracy.^56^ A Markov cohort model was selected over a microsimulation due to the availability of clinical evidence and MSAC preference.^57^ We model an average cohort and do not simulate individual patients. The Spanish analysis utilized a microsimulation to model cutaneous and extracutaneous response separately and included health states for stable and progressed non-responders. We modeled total complete or partial response collectively and combined stable and progressed non-responder health states into a single progressed health state to accommodate Australian-specific clinical evidence. Using a microsimulation would reduce the reliability of the result due to the small size of our clinical data sample.^56^ Our model structure permits the use of Australian-specific evidence, aligns with updates in best practice of reporting outcomes, and accurately represents disease progression and response to treatment.

Importantly, our analysis has been validated by MSAC as part of the health technology assessment process in Australia and had direct implications on healthcare policy and access. In July 2021, the MSAC recommended the public reimbursement of ECP for cGVHD, based in part on the findings of this economic evaluation. As part of the successful application, the MSAC suggested a respecified base case of a 5-year time horizon and the cost of rituximab removed from later line therapy may be reasonable, to align with published precedence and given rituximab was funded by state public payers rather than by the national public payer.^26^ This scenario produced an ICER of $48 764. We believe a time horizon of 10 years to be appropriate, given that treatment refractory cGVHD is a lifelong condition in which patients are likely to live with the disease for over 10 years, and the efficacy data applied in this evaluation is from registry studies which assessed patient outcomes over an 8-year period. Rituximab has since achieved an unrestricted listing in Australia, meaning it can now be routinely used as a later line therapy for cGVHD and is funded by the Australian public payer. Nonetheless, ECP was found to be cost-effective and approved by the MSAC, and subsequently listed on the MBS for the treatment of cGVHD.

This study has limitations related to the quality of clinical evidence, quality-of-life inputs, and treatment assumptions. Regarding the evidence, the nonrandomized design of the ECP evidence inherently carries a risk of potential bias. However, the 2 Australian patient registries were selected to inform the efficacy of ECP due to their high external validity and applicability to the Australian setting. These data were used to support regulatory and reimbursement approval in Australia and aggregate data used in this economic evaluation are publicly available.^26^ Complete and partial clinical skin response rates reported in this evidence were higher than that reported in the RCT evidence.^21^ This is likely attributed to differences in the definition of response from the 2006 clinical practice guidelines used in the RCT, compared with the Australian evidence, which used the 2014 NIH guidelines to assess overall or partial treatment response.^17,58,59^ The sensitivity analysis showed that the cost-effectiveness of ECP was moderately impacted by progression rate but remained cost-saving at the upper limit tested. Other nonrandomized evidence has also demonstrated ECP to have significant improvement in extracutaneous involvement and trial evidence published since the inception of the updated guidelines has shown similar complete or partial overall response rates.^20,22,24,25,48^ Overall, the efficacy of ECP and SoC applied in our evaluation is reflective of Australian practice and is similar to other published literature.

Our analysis used health state utility values from the Spanish cost-effectiveness analysis. The study and cited publications did not report how patient reported outcomes were mapped to health state utilities, which presents a limitation in the approach. Utility values decreased with progressively worse health states and met face validity as judged by Australian clinicians, thus were determined to be applicable to the modeled population. These utilities were also found to be comparable to those in a recently published literature review of quality of life associated with acute myeloid leukemia.^60^ This study mapped QLQ-C30 values to EQ-5D, which produced similar utility values for cGVHD (0.691) and no cGVHD (0.864) health states.^60^ Our sensitivity analysis demonstrated that utility values are not a driver of cost-effectiveness when aligned with the published evidence; thus, they are considered reasonable for our analysis.

The clinical manifestations of cGVHD are heterogenous, with choice of therapy largely dependent on the particular organ or site that is affected.^61^ Indeed, international guidelines state there is no consensus on the optimal choice of therapy for treatment refractory cGVHD.^17,18^ Mycophenolate mofetil, tacrolimus, and cyclosporine, which constitute second-line SoC options in this economic evaluation, were specific to the Australian healthcare setting and were selected based on clinical expert advice, availability on the PBS and precedence in Australian reimbursement decision making. The use of a survey to establish SoC is a limitation, as results may not be generalizable to all practice in Australia. However, noting the limited availability of published data, this was deemed the most practical method for establishing current Australian practice. Survey respondents treated approximately 250 cGVHD patients per year, corresponding to 88% of estimated cGVHD cases in Australia.^8^ As responses were received from most major cGVHD treatment centers, this demonstrates reasonable coverage of the Australian cGVHD population. There are several novel therapies currently being explored or being used for the treatment of cGVHD, including tyrosine kinase inhibitors, JAK1/2 inhibitors, proteasome inhibitors, and monoclonal antibodies. The survey indicated these were used infrequently in Australia at the time of conducting the analysis; however, they may be used in the future as access to these emerging therapies improves. The treatments considered in this analysis are included in international guidelines and treatment selection in this analysis is likely reflective of international cGVHD treatment.^3^

This cost-utility analysis demonstrates that ECP is cost-effective and should be considered as an alternate treatment option for steroid-refractory cGVHD in Australia. Compared with SoC therapies, ECP delayed use of expensive later-line therapies, reduced costs associated with disease manifestations, and improved quality of life. The listing of ECP on the MBS reflects the recognition as an effective treatment, which will lead to equitable access and support better outcomes for patients, whilst improving resource use for the public payer. Future research should investigate the long-term comparative efficacy of ECP vs novel therapies (ie, ruxolitinib and ibrutinib) and patient reported outcomes of treatment for cGVHD.

### Disclosures

F.D. and C.T. report receiving consulting fees from Mallinckrodt Pharmaceuticals; O.Z., F.G., and M.W. report payments/support as employees of Mallinckrodt Pharmaceuticals; N.H., S.L., and S.H. report receiving honoraria from Terumo BCT; and A.P. reports receiving consulting fees from Mallinckrodt Pharmaceuticals.

### Presentations

This economic analysis was presented to the Medical Services Advisory Committee (MSAC) which led to the public funding of ECP for cGVHD. Research related to our manuscript has also been presented at scientific conferences including Blood 2022 (the combined Annual Scientific Meeting of the Haematology Society of Australia and New Zealand, Australian and New Zealand Society of Blood Transfusion Thrombosis and Haemostasis Society of Australia and New Zealand), and the European Society for Blood and Marrow Transplantation 2022 Annual Meeting.

## Supplementary Material

Online Supplementary Material
