# Supplementary material for: Cost-Effectiveness of Extracorporeal Photopheresis in Patients With Chronic Graft-vs-Host Disease
Source: J Health Econ Outcomes Res. 2024 Feb 1;11(1):23–31. doi: 10.36469/001c.92028 (PMC10838062; doi:10.36469/001c.92028)

Journal of Health Economics and Outcomes Research

Online Supplementary Material

Cost-Effectiveness of Extracorporeal Photopheresis in Patients With Chronic Graft-vs-Host Disease.
JHEOR. 2024;11(1):*23*-31. doi:10.36469/jheor.2024.92028

Appendix: Treatment Survey to Determine Standard of Care

Figure S1: Markov Trace of the Extracorporeal Photopheresis Arm

Figure S2: Markov Trace of the Standard of Care Arm

This supplementary material has been provided by the authors to give readers additional information about their work.

This is an open-access article distributed under the terms of the Creative Commons Attribution 4.0 International License (CCBY-4.0). View this license's legal deed at http://creativecommons.org/licenses/by/4.0 and legal code at http://creativecommons.org/licenses/by/4.0/legalcode for more information.

Background information

1. Please complete the following details

- Name

- Hospital/Organisation

- State

- Post Code

- Qualification

- Years of experience treating cGVHD patients:

  ☐ 1-5
  ☐ 5-10
  ☐ 10-20
  ☐ 20-30
  ☐ 30+

- Number of cGVHD patients treated per year

- Number of newly diagnosed cGVHD patients treated each year

- Email address

Treatment pathways

PLEASE ASSUME ECP IS NOT AVAILABLE FOR USE WHEN ANSWERING THE FOLLOWING QUESTIONS:

2. For adult patients diagnosed with steroid-refractory or steroid-intolerant cGVHD what are the estimated proportions (in percent) of therapies listed below used in the **second-line** (after treatment with systemic steroids) setting?

- Mycophenolate mofetil: %
- Tacrolimus: %
- Sirolimus: %
- Ciclosporin: %
- Methotrexate: %
- Rituximab: %
- Ibrutinib: %
- Ruxolitinib: %
- Imatinib: %
- Interferon: %
- Other, please specify:

Drug name:

Proportion: %

3. For adult patients diagnosed with steroid-refractory or steroid-intolerant cGVHD what are the estimated proportions (in percent) of therapies listed below used in the **third-line** setting?

- Mycophenolate mofetil: %
- Tacrolimus: %
- Sirolimus: %
- Ciclosporin: %
- Methotrexate: %
- Rituximab: %
- Ibrutinib: %
- Ruxolitinib: %
- Imatinib: %
- Interferon: %

- Pulsed corticosteroids: [%]
- Other, please specify:

Drug name: [ ]

Proportion: [%]

4. Do you agree that ECP will be placed as a second-line therapy (i.e. used amongst other treatments listed in question 2)?

☐ Yes

☐ No

If no, please provide rationale:

[ ]

5. Do you agree that ECP will be used concomitantly with other second-line therapies?

☐ Yes

☐ No

If no, please provide rationale:

[ ]

Thank you for completing the survey.

Figure S1. Markov Trace of the Extracorporeal Photopheresis Arm

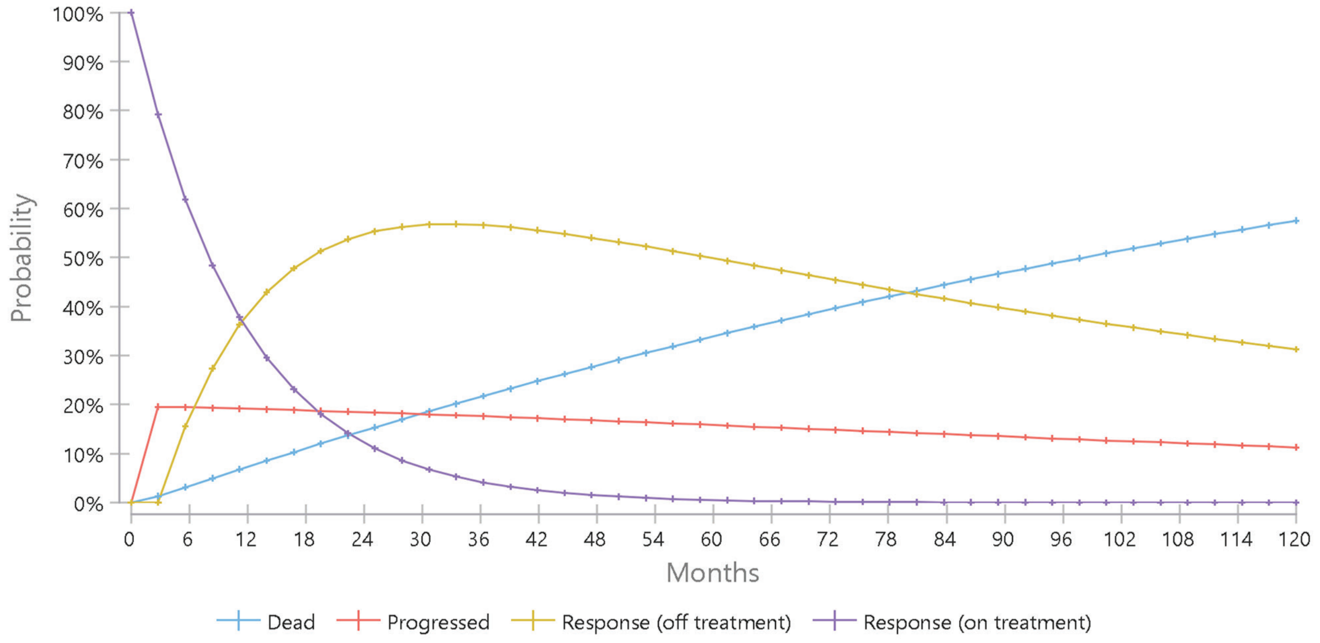

Figure S2. Markov Trace of the Standard of Care Arm

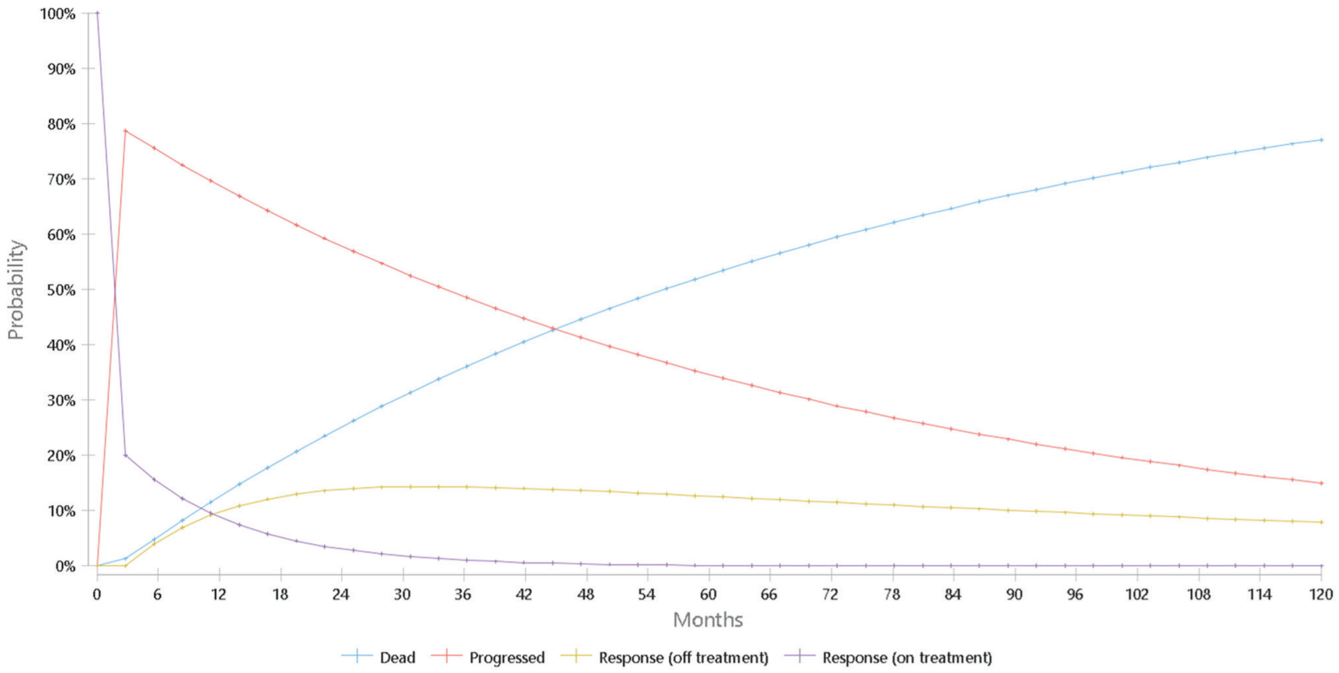

Supplement: Online Supplementary Material [file jheor_2024_11_1_92028_194132.pdf]
